# Global transcriptome dissection of pollen–pistil interactions induced self-incompatibility in dragon fruit (*Selenicereus spp.)*

**DOI:** 10.7717/peerj.14165

**Published:** 2022-11-01

**Authors:** Jun-cheng Li, Yulin Wang, Hong-fen Dai, Qingming Sun

**Affiliations:** 1 Institute of Fruit Tree Research, Guangdong Academy of Agricultural Sciences, Key Laboratory of South Subtropical Fruit Biology and Genetic Resource Utilization, Ministry of Agriculture and Rural Affairs, Guangdong Provincial Key Laboratory of Tropical and Subtropical Fruit Tree Research, Guangzhou, Guangdong, China; 2 School of Life Sciences, Guangzhou University, Guangzhou, Guangdong, China

**Keywords:** Dragon fruit, Self-incompatibility, Gametophyte self-incompatibility, S genes, Non-S genes

## Abstract

Self-incompatibility (SI) is a major issue in dragon fruit (*Selenicereus spp.*) breeding and production. Therefore, a better understanding of the dragon fruit SI mechanism is needed to improve breeding efficiency and ultimate production costs. To reveal the underlying mechanisms of SI in dragon fruit, plant anatomy, *de novo* RNA sequencing-based transcriptomic analysis, and multiple bioinformatic approaches were used to analyze gene expression in the pistils of the self-pollinated and cross-pollinated dragon fruit flowers at different intervals of time after pollination. Using fluorescence microscopy, we observed that the pollen of ‘Hongshuijing’, a self-incompatible dragon fruit variety (*S. monacanthus*), germinated on its own stigma. However, the pollen tube elongation has ceased at 1/2 of the style, confirming that dragon fruit experiences gametophyte self-incompatibility (GSI). We found that the pollen tube elongation *in vitro* was inhibited by self-style glycoproteins in the SI variety, indicating that glycoproteins were involved in SI. That is to say the female S factor should be homologous of S-RNase or PrsS (*P. rhoeas* stigma S factor), both of which are glycoproteins and are the female S factors of the two known GSI mechanism respectively. Bioinformatics analyses indicated that among the 43,954 assembled unigenes from pistil, there were six S-RNase genes, while 158 F-box genes were identified from a pollen transcriptomic dataset. There were no P. *rhoeas* type S genes discovered. Thus, the identified S-RNase and F-box represent the candidate female and male S genes, respectively. Analysis of differentially expressed genes (DEGs) between the self and cross-pollinated pistils at different time intervals led to the identification of 6,353 genes. We then used a weighted gene co-expression network analysis (WGCNA) to find some non-S locus genes in SI responses in dragon fruit. Additionally, 13 transcription factors (TFs) (*YABBY4, ANL2, ERF43, ARF2, BLH7, KNAT6, PIF3*, two *OBF1*, two *HY5* and two* LHY/CCA*) were identified to be involved in dragon fruit GSI. Thus, we uncovered candidate S and non-S genes and predicted more SI-related genes for a more detailed investigation of the molecular mechanism of dragon fruit SI. Our findings suggest that dragon fruit possesses a GSI system and involves some unique regulators. This study lays the groundwork for future research into SI mechanisms in dragon fruit and other plant species.

## Introduction

Dragon fruit (*Selenicereus spp.,* syn *Hylocereus spp.*) (Korotkova, Borsch & Arias, 2017) is the fruit of several cactus species indigenous to the South Americas. With its increasing popularity in the fruit industry, the cultivated areas of dragon fruit in mainland China have grown yearly, from 13,000 ha in 2015 to 40,000 ha in 2018 (Yi et al., 2015). Thus, dragon fruit has a high potential for commercial cultivation in the future because it is a high-value crop/product. Furthermore, dragon fruit blooms at night, which avoids high temperatures and low humidity during the day in their native habitat. However, flowering during the night also dramatically decreases the chances of pollination by most insects, as pollinators are primarily active during the day.

Like 60% of flowering plants, dragon fruit is also self-incompatible (SI) (Williams et al., 2014a). SI is a plant’s way of preventing self-fertilization. It is the strategy by which flowering plants prevent outbreeding sterility or inbreeding depression (Hegedűs, Lénárt & Halász, 2012). However, SI and a lack of pollinators complicate dragon fruit’s mating process. In wild populations, bats and moths help with pollination (Zimmerman, Montilla & Crossman, 2013). However, their assistance is insufficient in the prominent zones of agricultural production. Although there are several commercial self-compatible varieties of dragon fruit, the sweetness and taste of fruits produced by self-compatible varieties are inferior to SI varieties. Nevertheless, SI varieties still hold a significant market share due to their high fruit quality. Therefore, as the area under cultivation expands, the problem of dragon fruit SI became increasingly crucial for dragon fruit production. For dragon fruit SI varieties, hand pollination at night after blooming is currently the primary solution. However, this approach involves much work and can also be complicated by adverse weather. Thus, SI is the most significant impediment to dragon fruit propagation and fruit yield. Therefore, it is critical to investigate and reveal the SI mechanism.

Flowering plant reproduction begins with the mating of a male and female, producing progeny from an oosperm containing both parents’ genetic information (Wang & Köhler, 2017; Duan et al., 2020). Once pollen reaches the stigma, it typically germinates, prompting the pollen tube to elongate and release sperm. However, not all pollen will be accepted by the pistil, even in self-compatible plants. There are two major mechanisms of SI, heteromorphic self-incompatibility and homomorphic self-incompatibility. Males and females of the same genotype have no chance of meeting each other in plants that exhibit heteromorphic self-incompatibility due to their different floral morphology (Costa et al., 2017). The plants exhibiting a homomorphic self-incompatibility will experience a species-specific pollen interaction in the style that serves as a precursor to determining the fate of the pollen (Su et al., 2020) and leads to rejection of self-pollen. This interaction in eudicots evolved into two types of self-incompatibility: gametophyte self-incompatibility (GSI) and sporophytic self-incompatibility (SSI).

SSI has been found only in the Brassicaceae plant. The key determinants of SSI are male and female S genes, such as *S*-locus protein 11(*SP11*)/S cysteine-rich (*SCR*) and the *S receptor kinase* (*SRK*) gene (Abhinandan et al., 2021). The S-RNase-based GSI mechanism has the broadest taxonomic distribution. It is known to be determined by the male and female S genes such as SLF(S-locus F-box)/SFBB (S-locus F-box brothers) and S-RNase (Lee, Huang & Kao, 1994; Williams et al., 2014a). Currently, a similar mechanism has been found in Solanaceae (Williams et al., 2014b), Rosaceae (Qi et al., 2020), and Plantaginaceae (Meng & Sun, 2011). However, in *Papaver rhoeas* (*P. rhoeas*), there is another GSI system based on the male and female factors, the pollen S gene (PrpS) and stigma S gene (PrsS) (Wheeler et al., 2009).

In the most prevalent S-RNase-based GSI, a particular SI locus (S locus) houses one S-RNase and either one or a subset of SLF/SFBB gene(s) as female and male determinants, which are typically called S genes or S-locus determinants. S-RNase can interact with a subset of non-self SLFs and protect pollen RNA, while self-pollen RNA will be degraded (Ken-Ichi et al., 2010; Williams et al., 2014b). In addition to the well-studied S genes, non-S genes also regulate the sexual reproduction of self-compatible and self-incompatible species (Hegedűs, Lénárt & Halász, 2012). Non-S genes are the genes that are involved in the self-incompatibility response but are not located at the S locus. Compared to S genes, non-S genes have less certain position and functionality. Thus, they are more difficult to identify. To date, several non-S genes have been identified.

Among the non-S locus genes, HT-B is needed to halt pollen tube growth and is the primary source of SI loss for *Lycopersicon* (Kondo et al., 2002). The 120 kDa glycoprotein is required for S-specific pollen rejection in *Nicotiana* (Nathan Hancock, Kent & McClure, 2005). CUL1, a cullin1 protein, is also required for pollen function in SI of Solanum (Li & Chetelat, 2014). SSK1, the skp1-like protein, is required for cross-pollen compatibility in S-RNase-based SI (Sun & Kao, 2018). RBX1, a subunit of the SLF containing SCF complex, mediates the degradation of non-self S-RNase (Garcia-Valencia et al., 2017). Studies found that a non-S-locus F-box gene can break SI in diploid potatoes (Ma et al., 2021).

Because different SI mechanisms are essential in plant reproduction and differ between plant species, so they have recently received much attention. However, much of the SI mechanisms are still understudied, particularly in fruit trees, which require more research. Among the most important questions in these studies are how many genes/proteins are responsible for S-haplotype specificity, how many non-S locus genes are included in these pathways, and whether any species-specific genes and pathways are involved.

In the case of dragon fruit, very little is known about its SI mechanism, particularly on the molecular level. A recent study has concluded that three species of Cactaceae exhibit the most prevalent type of S-RNase-based GSI (Ramanauskas & Igić, 2021). Our observations of dragon fruit pollen tube elongation in either self or cross-pollinated pistils also revealed that dragon fruit’s SI mechanism belongs to GSI. We further depicted that the progenies’ segregation of SI/SC crossing groups is incongruent with Mendel’s genetic law. These findings infer that the SI mechanism of dragon fruit could be much more intricate and complex than the S-RNase based system, thus necessitating the identification of additional potential SI factors. Identifying novel regulatory factors and dissecting the SI mechanism in fruit trees is challenging. High-throughput transcriptome sequencing, which generates a large amount of data, combined with bioinformatics and systems biology approaches to predict possible gene functions, is beneficial in this case. Coupling it with co-expression analysis to correlate genes of unclear functions with biological processes, prioritize candidate genes, or determine transcriptional regulatory programs from a systematic perspective is tremendously helpful (Van Dam et al., 2017). Using the combination of these approaches to investigate the molecular mechanism of dragon fruit SI allowed us to discover previously unknown dragon fruit SI-related genes. Our findings broaden our understanding of self-incompatibility in dragon fruit and provide a foundation for future studies.

## Material and Methods

### Plant materials

In this work, we used the Hongshuijing and Dahong dragon fruit varieties, which are well-known red flesh varieties with a high market value in mainland China. Hongshuijing is a self-incompatible variety that needs hand-pollination when blooming. However, it produces higher quality fruits. On the other hand, the Dahong variety is self-compatible and does not require hand-pollination. As a result, it is more popular on farms due to easier production. However, its fruit is of relatively low quality. All plants used in this study were grown and preserved in a greenhouse at the Institute of Fruit Tree Research, Guangdong Academy of Agricultural Science, China, ready to be shared with any researcher.

### Hand pollination

In the self-pollination experiment, 120 well-developed Hongshuijing flowers were randomly selected and bagged at 8:00 am on a clear day. Then, the bagged flowers were manually shaken at about 8:30 pm to promote self-pollination.

In the cross-pollination experiment, 120 well-developed Hongshuijing flowers were randomly selected at 8:00 am on a clear day, removed the anthers, and bagged. The pollen of Dahong as the pollination variety was collected after blossoming at 8:30 pm, brushed on the emasculated stigma of Hongshuijing for cross-pollination. Then, the cross-pollinated flowers were bagged again.

### Fluorescence microscopic observation of pollen growth in pistil

Self and cross-pollinated Hongshuijing flowers at 0, 12, 24, 36, 48, and 72 haps (hours after pollination) were collected. For each treatment, five flowers were sampled for each time point. FAA (50% alcohol: formalin: acetic acid = 85:10:5) was used to glue the pistils. Fluorescence microscopic observation was conducted as follows:

(1) The pistils were kept in FAA (50% alcohol: formalin: acetic acid = 85:10:5) at room temperature for 48 h before being stored at 4 °C.
(2) The pistils were rinsed with distilled water and immersed in 2 M NaOH for 1 h at 45 °C, then neutralized with glacial acetic acid for 30 s before being rinsed three times with distilled water.
(3) The pistils were dyed with 0.1% aniline blue (1 g aniline blue was dissolved with 1000 mL of pre-made K_3_PO_4_ solution at a concentration of 0.1 M) in the dark for 12 h.
(4) The sampled pistils were transversely cut into 1-cm-long segments and stored in order. Then, the segments of pistils were carefully slit longitudinally as pollen tubes clustered in the transmitting tissue. Finally, the transmitting tissue was viewed under a fluorescent microscope (ZEISS Axioplan 2; ZEISS, USA) at a wavelength of 360 nm after being separated and squished.

### Effects of glycoproteins on *in vitro* pollen tube elongation

Nine well-developed flowers of Hongshuijing were randomly selected at 8:00 am on a clear day, and anthers were removed and bagged. The style of each flower was collected after blooming at 8:30 pm and used in protein extraction. Three styles were combined in one sample, and the experiment was conducted in three replicates. Concurrently, the pollen of two varieties, Hongshuijing and Dahong, were collected separately. For each dragon fruit variety, the pollens of three flowers were mixed as a sample, and the experiment was conducted in three replicates. Total soluble proteins were extracted from the styles of Hongshuijing as described in the protocol of the plant total protein extraction kit (Sangon, Shanghai, China). Glycoproteins were separated from the total soluble proteins using the FOCUS™ Glycoprotein kit (Sangon, Shanghai, China), following the manufacturer’s instructions. The soluble proteins without glycoproteins collected from the style were named glycoproteins-free protein fractions.

The pollen of Hongshuijing (self-incompatible) and Dahong (cross-compatible with Hongshuijing) were cultured in a liquid medium (20% sucrose + 300 mg/L H_3_BO_3_ + 100 mg/L Ca(NO_3_)_2_.4H_2_O + 300 mg/L KNO_3_ + 300 mg/L MgSO_4_.7H_2_O under 28 °C, pH 7.0) supplemented with total soluble proteins sample, isolated glycoproteins, or the total proteins fraction from which glycoproteins were removed (glycoproteins-free fraction), respectively. The liquid medium without any extra protein added was used as control. The protein concentration in either of the protein samples described above was10 ng L^−1^. For *in vitro* germination experiment, 0.1 g of pollen grain was placed in a five mL liquid medium and incubated at a temperature incubator (28 °C and RH80%) for 12 h, and the pollen tube growth was investigated under a microscope. For each sample, three fields were observed, including no less than 30 pollen grains. The experiment was conducted in three replicates.

### Sample preparation and sequencing

A total of 21 pistil samples from seven treatments were used in this experiment. The 21 samples consisted of the 12, 24, and 36 h after self-pollination samples, the 12, 24, and 36 h after cross-pollination samples, and the control sample (without pollination). Five pistils from distinct plants were combined in one sample for each treatment. The experiment was conducted in three replicates. All these samples were subjected to RNA-seq and qPCR analysis. Total RNA was extracted with CTAB plus using an OMEGA Plant RNA Isolation kit according to the protocol described by Ouyang et al. (2014). The library construction and sequencing were conducted using a standard protocol by Novogene (Beijing, China).

### DEG identification and functional annotation

The raw data were filtered to remove adapter or low-quality sequences and assembled by the Trinity pipeline (Haas et al., 2013). The transcripts per million (TPM) approach was used to calculate unigene expression (Wagner, Kin & Lynch, 2012). The differential expression gene (DEG) analysis was performed using the R package “edgeR” 3.36.0 with the following thresholds: *q*-value 0.05 and |log2(foldchange)| > 1.5 (Robinson, McCarthy & Smyth, 2010). *q*-value was adjusted *p*-value using Benjamini and Hochberg’s approach (Benjamini & Hochberg, 1995). Power analysis was conducted with the R package RNASeqPower (Therneau & Stephen, 2014). The following databases were used to determine functional annotations: NCBI non-redundant (Nr) protein sequences, NCBI non-redundant nucleotide (Nt) sequences, protein family (Pfam), Clusters of Orthologous Groups of proteins (KOG/COG), Swiss-Prot, a manually annotated and reviewed protein sequence database, KEGG Ortholog (KO) database, and Gene Ontology (GO).

### Co-expression analysis

A weighted gene co-expression network analysis (WGCNA) was used in this study (Langfelder & Horvath, 2008). The input expression matrix included all expression data of DEGs from the pistil RNA-Seq. This R program divides the DEGs into multiple modules (gene sets) based on direct and indirect gene correlations. The systematic strategy, which included GO and KEGG enrichment analyses, was used to investigate the functions of these gene sets. The significance cutoff of GO and KEGG enrichment analysis is *q*-value (false discovery rate adjusted *p*-value) <0.05. The correlation matrix generated by the WGCNA was used to build the co-expression network. The network consisted of the gene connections and their 50 most correlated genes. Cytoscape v3.8.0 was used to visualize the network, and the nodes were ordered using the yFiles Layout Algorithms (Tuikkala et al., 2012).

### Real-time Quantitative PCR (qPCR) analysis

In total, 20 differentially expressed genes identified in RNA-seq datasets were randomly selected, and their expression was confirmed by qPCR. The qPCR was conducted using the SYBR Premix ExTaqII Kit (Takara) with the ABI StepOne Real-Time PCR system (Applied Biosystems, San Mateo, CA, USA). The thermal cycling conditions were as follows: an initial denaturation of 95 °C for 30 s, followed by 40 cycles of denaturation, annealing, and elongation (95 °C for 5 s and 60 °C for 30 s), then a melt-curve step (0.3 °C/s, from 60 to 95 °C). The relative expression level of each gene was calculated *via* the 2^−ΔΔCt^ method (Livak & Schmittgen, 2001) with three biological replicates. In addition, *actin* and *EF1- α* were used as reference genes (Chen et al., 2019).

## Results

### Pollen growth in pistil *in vivo* and effects of style glycoproteins on pollen tube elongation *in vitro*

**Figure 1:**
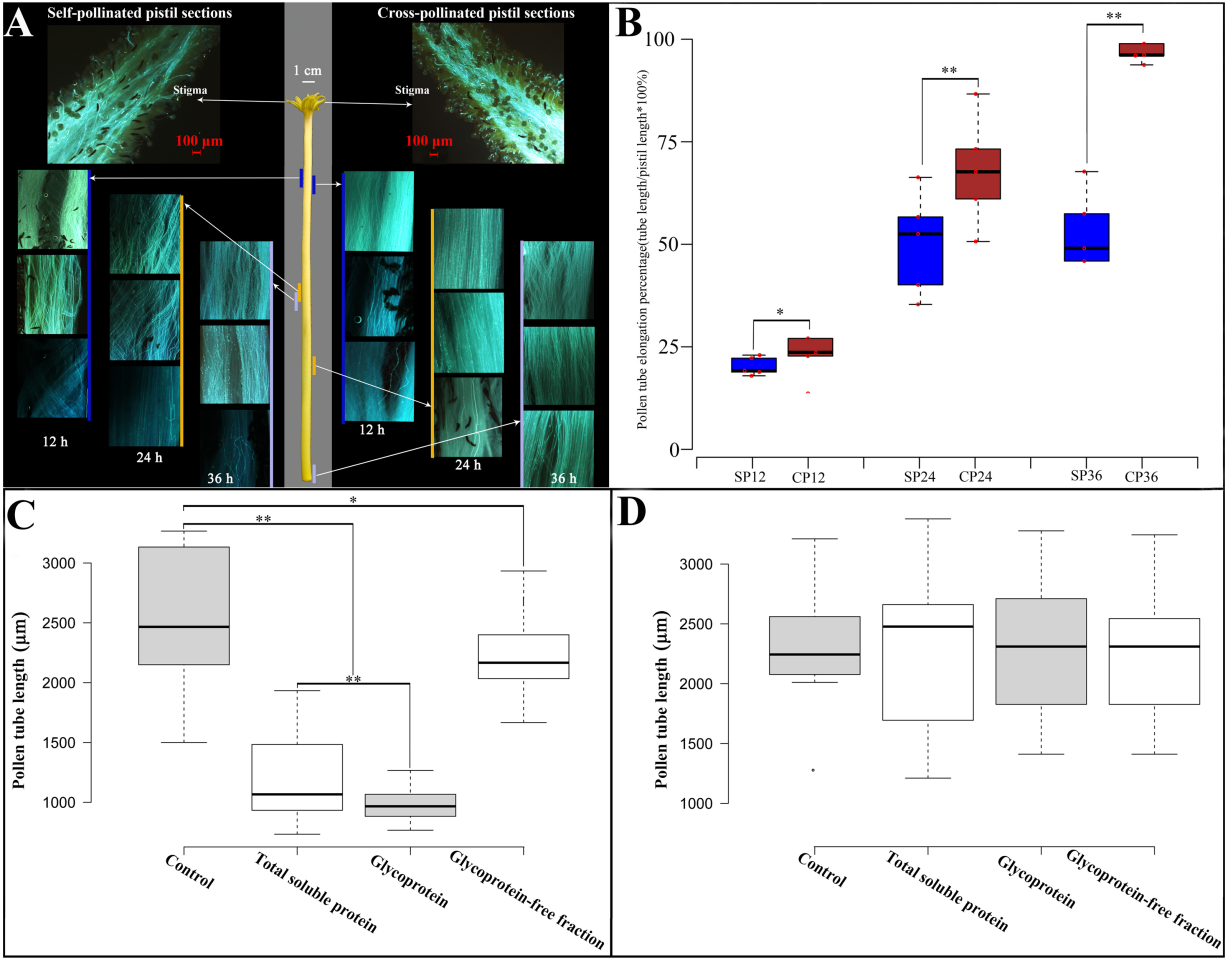
Pollen growth in pistil *in vivo* and effects of style glycoproteins on pollen tube elongation *in vitro*. (A) Time line of pollen germination on stigmatic surface and elongation of pollen tubes in style using fluorescent microscopy. Middle panel depicts the pistil of Hongshuijing (scale bar = one cm), left panel corresponds to self-pollinated pistil sections (scale bar = 100 um), right panel corresponds to cross-pollinated pistil sections (scale bar = 100 um). Lines of different colors (blue, brown and gray) along with the left and right sides of the style represent where the pollen tube reached style at different time points. (B) Boxplots of pollen tube elongation rates at different time points after self-pollinated (SP) and cross-pollinated (CP). SP12/CP12, SP24/CP24, SP36/CP36 mean 12, 24, 36 hours after either self or cross pollinated, respectively. C and D: Pollen tube elongation of Hongshuijing (panel C) and Dahong (panel D) *in vitro* using different treatments. Control, medium without any extra protein added. Total soluble protein, medium supplemented with total soluble proteins derived from the style of Hongshuijing. Glycoprotein, medium supplemented with isolated glycoproteins from the style of Hongshuijing. Glycoproteins-free fractions, liquid medium supplemented with glycoproteins-free protein fractions from the pistil of Hongshuijing. The “*” or “**” on the figure means significant (*p*-value ≦ 0.05) or highly significant (*p*-value ≦ 0.01) different with *T*-test, respectively.

To observe *in vivo* pollen tube elongation in the pistil of Hongshuijing, we conducted self-pollination and cross-pollination of Hongshuijing with Dahong pollen. The pollen grain can germinate on the stigma of self- and cross-pollination (Fig. 1A, Table S1). After self-pollination, the pollen tube elongated to 17.5% of style after 12 h, reached the middle parts (48.5%) of style after 24 h, and then 50.2% and 50.4% of style after 36 and 48 h, respectively. It demonstrated that the pollen tube elongated to the middle parts of the style and almost ceased extension at 24 h after self-pollination. However, in cross-pollination, the pollen tube elongated to 19.9% of style after 12 h, extended to nearly 2/3 (69%) of the style length after 24 h, and then 99.3% and 100% of style after 36 and 48 h, respectively.

It is indicated that the elongation of the self-pollinated pollen tube in style was significantly slower than that of the cross-pollinated pollen tube at different time intervals (Fig. 1B). The pollen tube almost ceased elongation at 24 h after self-pollination, and the pollen tube in cross-pollination entered the ovary after 36 h. This result displays that dragon fruit exhibits gametophytic self-incompatibility (GSI).

Based on the hypothesis that S glycoprotein is the leading participant in GSI, we performed an *in vitro* pollen germination test using a germination medium supplemented with total soluble proteins, glycoproteins’ fraction isolated from total soluble proteins, and a glycoproteins-free fraction of total soluble proteins derived from the style of Hongshuijing. We discovered that the pollen tube elongation of Hongshuijing (self-incompatible) was sensitive to the total soluble proteins and glycoproteins but tolerated the non-glycoproteins compared to the control (Fig. 1C). However, the pollen tube elongation of Dahong (cross-compatible with Hongshuijing) was not affected by the three protein treatments (Fig. 1D).

The results of *in vivo* pollen tube elongation and the effect of the style glycoproteins on *in vitro* pollen tube growth demonstrated that dragon fruit exhibits the GSI, and glycoproteins are the players in the mechanism of self-incompatibility. This is consistent with the findings that the female S determinants are glycoproteins in the two known GSI mechanisms, either the S-RNase-based type or the poppy type of GSI.

### Transcriptomes analysis of self- or cross-pollinated pistils

We sequenced RNA from pistil tissue from 21 different samples to investigate and compare the global transcriptome dynamics in Hongshuijing pistils in response to self and cross-pollination. These samples included control (without pollination), SP12, SP24, and SP36, CP12, CP24, and CP36 (corresponding to 12, 24, and 36 h after self or cross-pollination, respectively). The transcriptome of Hongshuijing pollen was investigated individually. A total of 155.83 GB of clean data was retrieved from 21 samples, with each sample containing at least 6.51 GB of clean data with a Q30 quality score ≥ 95.21% (Table S2).

**Figure 2:**
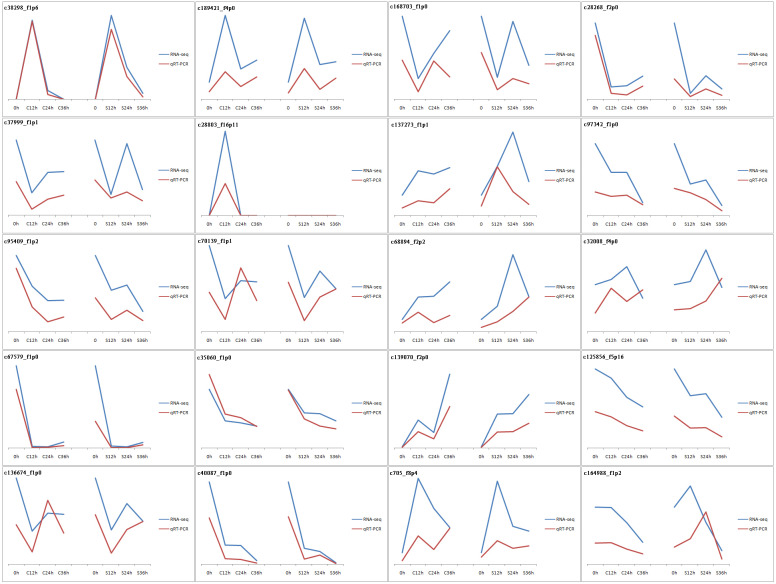
Validation of RNA-seq results by qPCR. 20 genes were used in this experiment. The results are displayed by line charts. RNA-seq data are depicted as blue lines, qPCR results are depicted as the red lines.

The vast majority of the treatments were found to be highly reproducible. Only one CP36 sample replicate did not cluster with the other two samples (Fig. S1). In total, 43,954 unigenes were evaluated with an N50 of 1586 bp, and the guanine-cytosine (GC) content was 49.20%. The unigene length ranged from 201 to 5869 bp with an average length of 1,430 bp, of which 67.12% (29,503) were longer than 1,000 bp. The 42,194 unigenes out of the total 43,954 had functional annotations in the known database (Table 1), 91.8% of the annotated unigenes with *E*-value smaller than 10^−30^ (Fig. S2), and the final reference transcriptome is 61.2 Mb (unigene of dragon fruit pistil, average sequencing depth =108). Our previously uploaded pollen transcriptome data on NCBI (PRJNA822018) was also used for male S factors identification in this study. To validate the accuracy of our RNA-seq data, qPCR was used to verify the randomly selected 20 DEGs (the sequence and corresponding primers are listed in Table S3). Our results confirmed that the expression patterns of these genes were very similar to those found in RNA-seq (Fig. 2), confirming that our RNA-seq expression data were highly reliable and can be used for subsequent experimental analysis.

**Table 1:** Summary of unigene assembly and annotation.

	Pistil	Pollen
Total Unigene	43,954	41,395
Average unigene Length (bp)	1,430	819
Mediam unigene length (bp)	1,207	646
N50 (bp)	1,586	977
Smallest unigene length (bp)	201	201
Largest unigene length (bp)	5,869	6,477
200–500 bp	15	13,440
500–1,000 bp	14,436	17,530
1,000–2,000 bp	20,445	8,534
>2,000 bp	9,058	1,891
Annotated Unigene	42,194 (95.99%)	30,575 (73.86%)

### Identification of DEGs and cluster analysis

**Figure 3:**
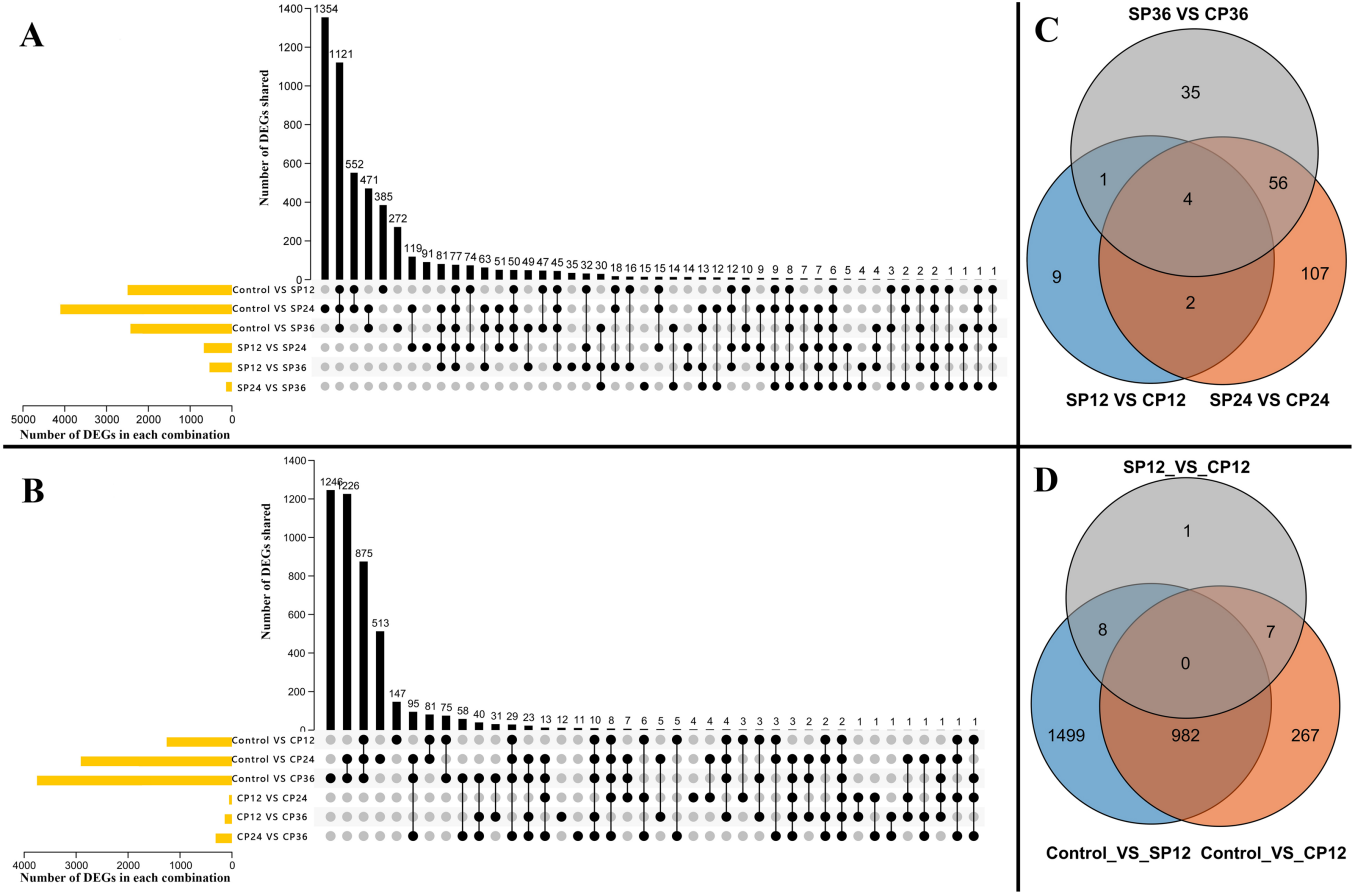
Distribution of the DEGs. (A) DEGs across six pairwise comparisons of the self-pollinated samples (control *vs* SP12, control *vs* SP24, control *vs* SP36, SP12 *vs* SP24, SP12 *vs* SP36, and SP24 *vs* SP36). Yellow bars in they-axis represent the total number of DEGs in each parwise combination. Black bars in the *x*-axis represent the number of DEGs shared across pairwise combinations connected by the dark dots in the body of the plot. (B) DEGs across six pairwise comparisons of the cross-pollinated samples (control *vs* CP12, control *vs* CP24, control *vs* CP36, CP12 *vs* CP24, CP12 *vs* CP36, and CP24 *vs* CP36). Yellow bars in they-axis represent the total number of DEGs in each parwise combination. Black bars in the *x*-axis represent the number of DEGs shared across pairwise combinations connected by the dark dots in the body of the plot. (C) Common and special DEGs between pistils of self- and cross-pollinated at 12 h, 24 h, and 36 h after pollination. The 214 common DEGs in total in this plot is the rejection related gene set. (D) Common and special DEGs cross three pairwise comparisons (control *vs* SP12, control *vs* CP12, and SP12 *vs* CP12). The shared 982 DEGs in total in this plot are the recognition related gene set.

The power analysis revealed that fold change (FC) >=1.5 could be consistently detected with a power of 0.9 and a false discovery rate (FDR) of 0.05 with a sample size of 7 when accounting for average sequencing depth and biological variance (Fig. S3). A total of 6,353 significant differentially expressed genes (DEGs), including 58 transcription factors (TFs), were identified from 15 pairwise comparisons. These included six comparisons of the self-pollinated samples (Fig. 3A, control *vs.* SP12, control *vs.* SP24, control *vs.* SP36, SP12 *vs.* SP24, SP12 *vs.* SP36, and SP24 *vs.* SP36), six comparisons of the cross-pollinated samples (Fig. 3B, control *vs.* CP12, control *vs.* CP24, control *vs.* CP36, CP12 *vs.* CP24, CP12 *vs.* CP36, and CP24 *vs.* CP36), and three comparisons of the self and cross-pollinated samples (Fig. 3C, SP12 *vs.* CP12, SP24 *vs.* CP24, and SP36 *vs.* CP36). In the case of self-pollinated pairwise comparisons, control *vs.* SP24 had the most DEGs (Fig. 3A). However, control *vs.* CP36 yielded the highest number of DEGs in CP comparisons (Fig. 3B). Furthermore, SP24 *vs.* CP24 exhibited more DEGs than the other two comparisons (Fig. 3C). These findings correlated with the result of the SP experiment which depicted that the pollen tube ceased elongating after around 24 h (Fig. 1A and 1B), indicating that more genes have been up or down-regulated to suppress the pollen tube elongation.

SI is the consequence of a complex interplay of biological processes, most of which are unknown in dragon fruit. To better understand these processes, we artificially divided them into two subprocesses: pollen recognition and rejection. We discovered that the pollen recognition occurred before 12 haps because the pollen tube length in SP treatment was already shorter than in CP treatment (Fig. 1A and 1B). In pairwise comparisons, 982 DEGs were common between control *vs.* SP12 and control *vs.* CP12 (Fig. 3D). We hypothesized that these pollen recognition-related genes would be included in the 982 DEGs because recognition occurs in both SP and CP treatment. Furthermore, the expression profiles of these 982 DEGs were similar, with expression values increasing or decreasing in both SP and CP samples from 0 to 12 hap. Following recognition, the stigma can assess whether the pollen tube is from self-pollen and determine whether to reject or accept it. Pollen tube rejection is a continual interaction between pollen and pistil until the pollen tube stops elongation (Chen et al., 2018). It is unclear when exactly the rejection occurs, but it is certain that it will only happen in SP pistils. Therefore, most of the rejection-related genes will be differentially expressed in SP pistils and thus will be included in the 214 DEGs that existed between SP and CP (Fig. 3C). Therefore, the two important DEGs sets, the 982 DEGs as candidate pollen recognition-related genes and the 214 DEGs as potentially rejection-related genes were identified.

Using WGCNA analysis with the soft threshold of 12 (Fig. S4 A, the approximate scale-free fit index can be attained when the soft-thresholding power *β* was set as 12) all 6,353 DEGs between the pistils at different intervals of time after self- or cross-pollination were assigned to ten modules. These modules are depicted in different colors: black, blue, brown, green, magenta, pink, red, turquoise, yellow, and grey. The grey module, which consisted of 88 genes, represented the group of genes that could not be classified. (Table S4; Fig. S4B). The biggest module depicted in turquoise contained 2483 genes, and the smallest module depicted in magenta contained 74 genes. Of the two crucial DEGs sets, in the 214 DEGs gene set related to pollen recognition, 132 (61.7%) of the 214 genes were assigned to the green module, which contains a total of 461 genes, and in the 982 DEGs gene set related to rejection, 534 (54.4%) of the 982 genes were assigned to the turquoise module, which contains the total of 2,483 genes (Table S5). A module is a collection of genes that work together to form a single or a series of interconnected processes. We hypothesized that the green and turquoise modules are the other two DEG sets containing SI-related genes. Therefore, four DEGs sets, the pollen rejection related 214 DEGs (Fig. 3C, the DEGs between CP12 and SP12, CP24 and SP24, CP36 and SP36), the recognition related 982 DEGs (Fig. 3D, the shared DEGs between control and CP12, control and SP12), the green module and the turquoise module (two of the modules generated by WGCNA with a large proportion of overlap with the 214 and 982 DEGs sets), were identified through two different approaches. Furthermore, the identified gene set overlap, the 132 DEGs overlapped between the 214 DEGs and the green module, and the 534 DEGs overlapped between the 982 DEGs and the turquoise module were narrower gene sets potentially involved in SI.

### GO and KEGG functional enrichment analyses of the key DEGs sets

**Figure 4:**
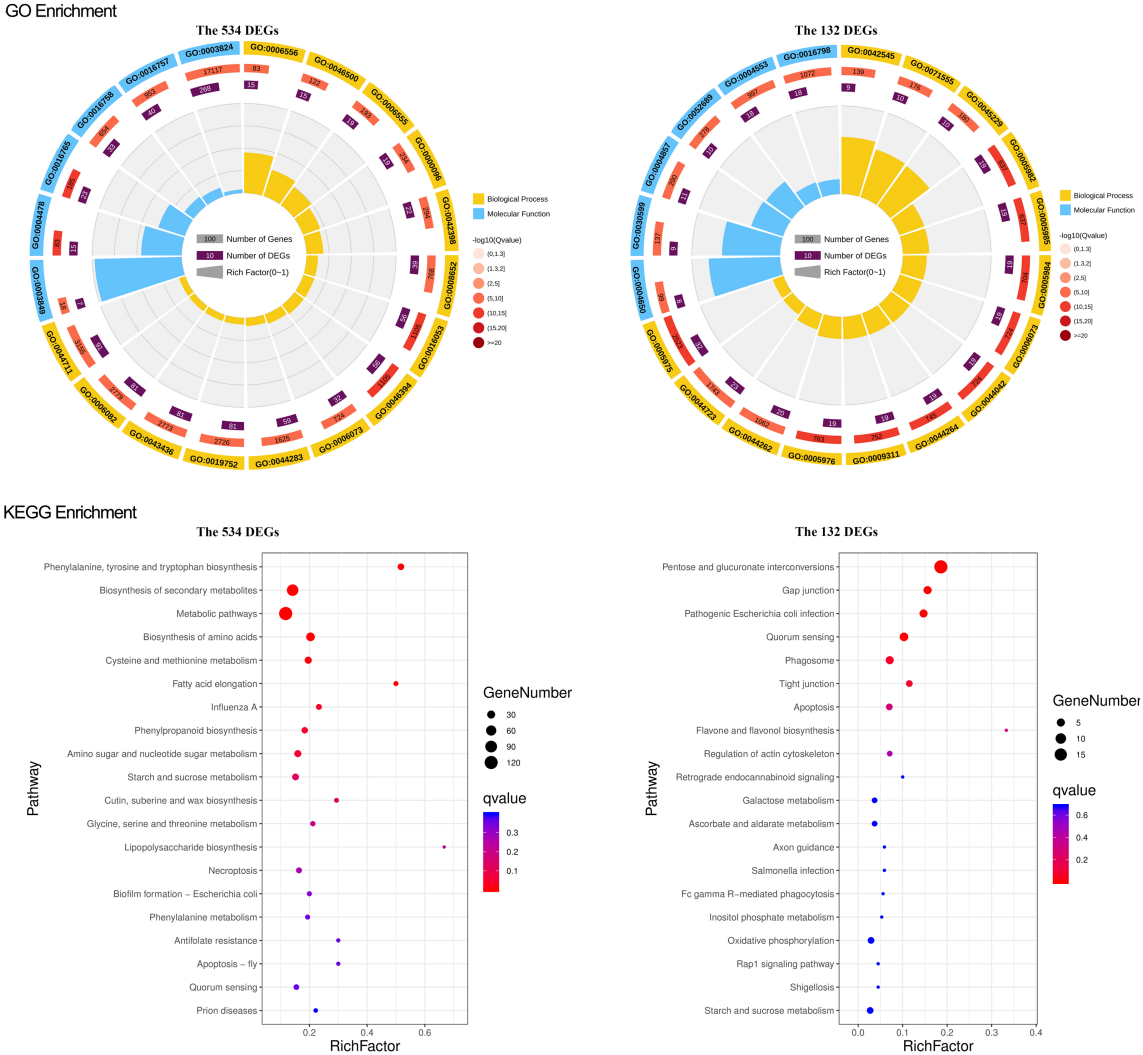
GO and KEGG enrichment of the overlapped 534 DEG and 132 DEGs. For GO enrichment, the first circle: the GO term of the first 20 enriched terms. Different colors represent different ontologies (blue for biological process, yellow for molecular function); the second circle: the number of genes in the GO term of the background and *Q* value of the enrichment. The more genes the longer the bar, the smaller the *Q* value and the redder the color, most of the −log10 (*Q* value) is between 10 and 15; the third circle, enriched gene proportion bar chart in purple, the more genes the longer the bar; the fourth circle, enrich factor value of each GO term (the number of DEGs is divided by the number of genes in the background in the GO term, each grid of the background grid line represents 0.1). For KEGG enrichment, the first 20 enriched pathways were shown in the figure; the dot’s size and color represent the gene number and *Q* value enriched in each pathway.

To study what could be the functions of the identified gene sets, we conducted a general survey with GO and KEGG enrichment. The results were particularly pronounced for the two narrowed-down gene sets, the overlapped 132 DEGs and 534 DEGs. For the overlapped 132 DEGs, there were 51 GO terms, including 24 for biological process, 23 for molecular function, and four for cellular components significantly enriched. Most of the genes in the biological process are involved in the carbohydrate metabolic process. The molecular function terms are mainly related to hydrolase activity and catalytic activity. The cellular component genes were located in the cell wall, external encapsulating structure, cell periphery, and microtubule (Fig. 4). KEGG pathway and enrichment analysis demonstrated four significantly enriched pathways, mainly in the pathways of pentose and glucuronate interconversions (Fig. 4).

For the overlapped 534 DEGs, there were 216 GO terms, including 156 for biological process, 55 for molecular function, and 7 for cellular components significantly enriched. The enriched GO terms mainly included small molecule metabolic processes, carboxylic acid metabolic process, oxoacid metabolic process, transferase and oxidoreductase activity, extracellular region, apoplast, and cell wall (Fig. 4). The KEGG pathway and enrichment analysis revealed that nine pathways were significantly enriched, primarily in the pathways of secondary metabolite and amino acid biosynthesis, cysteine, and methionine metabolism (Fig. 4). GO and KEGG functional enrichment analyses of the other 4 DEGs sets, 214, 982, the green module, and the turquoise module, are depicted in Figs. S5 and S6.

### Identification of S genes and non-S locus genes

Searching for the homologous genes in other species is the standard method to identify the candidate genes. Based on the most common S-RNase-based GSI mechanisms, six candidates of S-RNase were identified in the Hongshuijing pistil database, and 158 F-box genes were identified in the Hongshuijing pollen database (Table S6). However, we did not find any homologous genes of PrsS and PrpS, so we exclude the possibility of *P. rhoeas* type SI in dragon fruit. As a result, we hypothesized that the identified S-RNase and F-box genes are candidate female and male S genes, respectively.

In addition to well-known S genes, non-S locus genes have also been discovered to participate in GSI (Zhang et al., 2017; Su et al., 2020; Ma et al., 2021).To simplify the transcriptome gene expression data, we used the principle of data dimensionality reduction. A gene co-expression analysis was carried out based on the TOM matrix generated by WGCNA analysis. A gene was designated as a node in each network in our analyses, and its co-expression with other genes was represented as lines (edges). The degree of connectedness of a node was defined as the number of genes associated with it. Hub genes, which act as representations of a gene set, had the highest degree of interconnectedness. Here, the top 50 genes of the co-expression network were defined as hub genes (Tables S7 and S8). By querying the Pearson’s Correlation Table (Degree of Freedom = 20, Pearson’s correlation >0.536800), we found that all correlations in the network reached a highly significant level (*p* < 0.01).

The network hub is defined by a mathematical algorithm rather than a biological method. However, the biological significance can be speculated based on functional gene annotations. Among the first 10 hub genes of co-expression network for the 534 DEGs, seven of them may directly or indirectly be involved in SI response (Table S7), which were annotated as F-box (c37611_f1p1), U-box E3 ubiquitin ligase (c104651_f1p2) (Kim et al., 2021), pistil-specific extension-like proteins III (PELPIII) (c28784_f2p0), 120 kDa (c96768_f1p3) (Schultz et al., 2002; Hegedűs, Lénárt & Halász, 2012), serine/threonine-protein kinase isoform X2 (c62321_f1p0) (Nie et al., 2019), cysteine-rich receptor-like protein kinase (c106266_f2p1) and aquaporin (c27095_f28p9) (Ikeda et al., 1997). The other three hub genes, YABBY4 (c30021_f1p0), which is involved in seed abortion in *Vitis vinifera L*. and elongates the pistil abnormally in tomatoes when overexpressed (Massonnet et al., 2020), xyloglucan galactosyltransferase XLT2 (c131771_f1p0), which is involved in plant cell wall polysaccharide xyloglucan biosynthesis (Schultink, 2013), and ferredoxin-like protein (c1725_f3p0) which functions in stress resistance (Huang et al., 2020), were among the 10 hub genes that had no obvious connection to known function in SI response.

For 132 DEGs, six of the first 10 hub genes may directly or indirectly be involved in SI response (Table S8). Those were annotated as beta-galactosidase (c161600_f1p9) (Wanyi et al., 2019), pectinesterase (c127787_f1p9, c70692_f1p1, c99737_f1p2) (Wanyi et al., 2019), 120 kDa (c28368_f3p4) (Lin et al., 2014) and arabinogalactan protein (c9808_f1p3) (Pereira, Lopes & Coimbra, 2016). Interestingly, the other four hub genes, c1947_f8p6, c7984_f1p0, c103690_f1p1, and c205802_f1p2, were not annotated in any databases (Table S9). However, we cloned them from dragon fruit pistils and fully verified their sequences. We hypothesized that they are new genes from dragon fruit and may participate in the GSI regulation.

**Figure 5:**
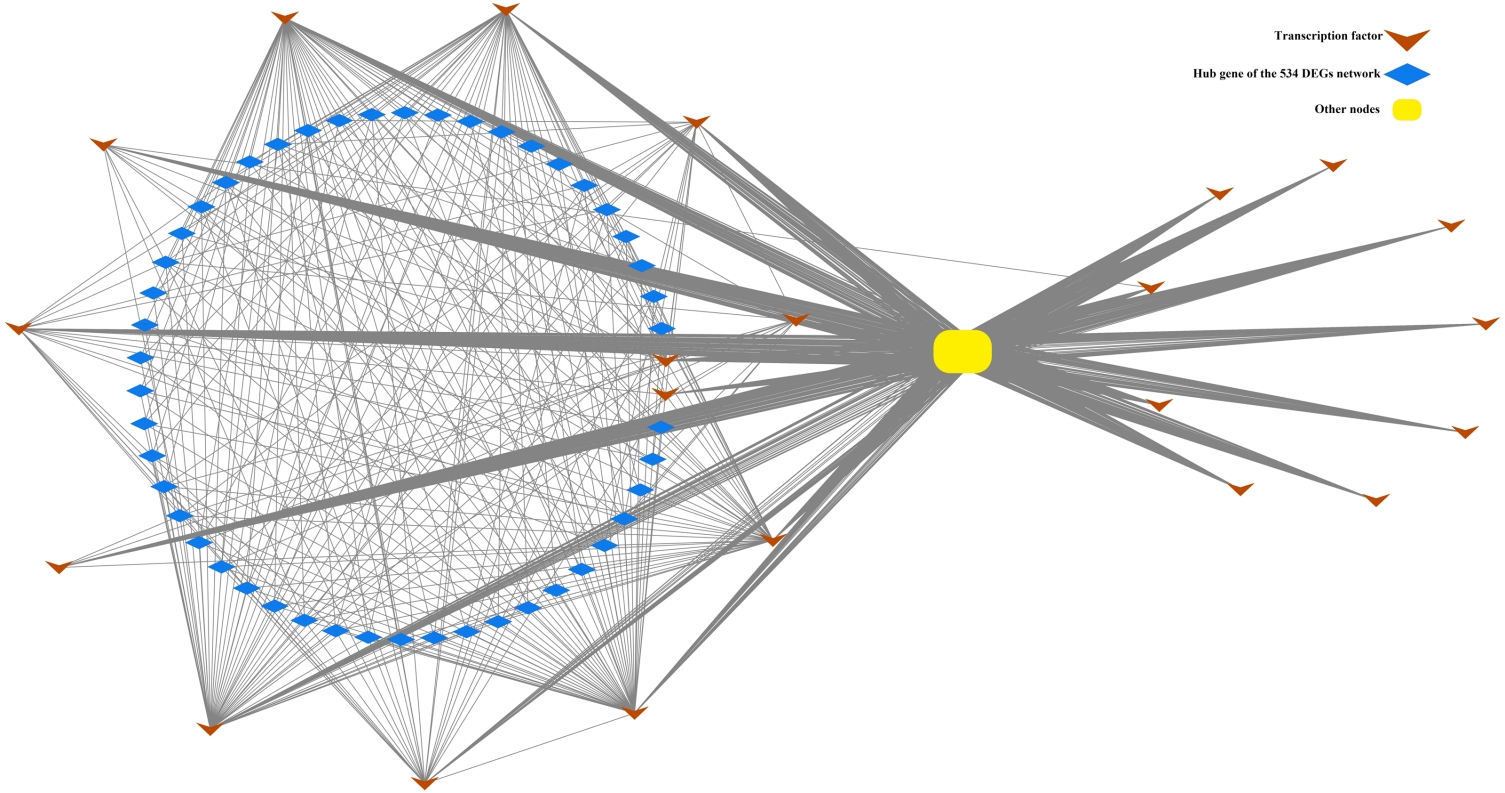
Correlation of TF22 and the top 50 hub genes of the overlapped 534 DEG network. This network consists of nodes (blue rhombuses, yellow square and brown arrows) as well as lines that represent genes and intergenic correlations. The red rhombuses and blue square represent the hub genes and other genes in network 534 DEGs, respectively. The green arrows represent the transcription factors of TF22. The 50 hub genes and 22 TFs share 2 genes.

**Figure 6:**
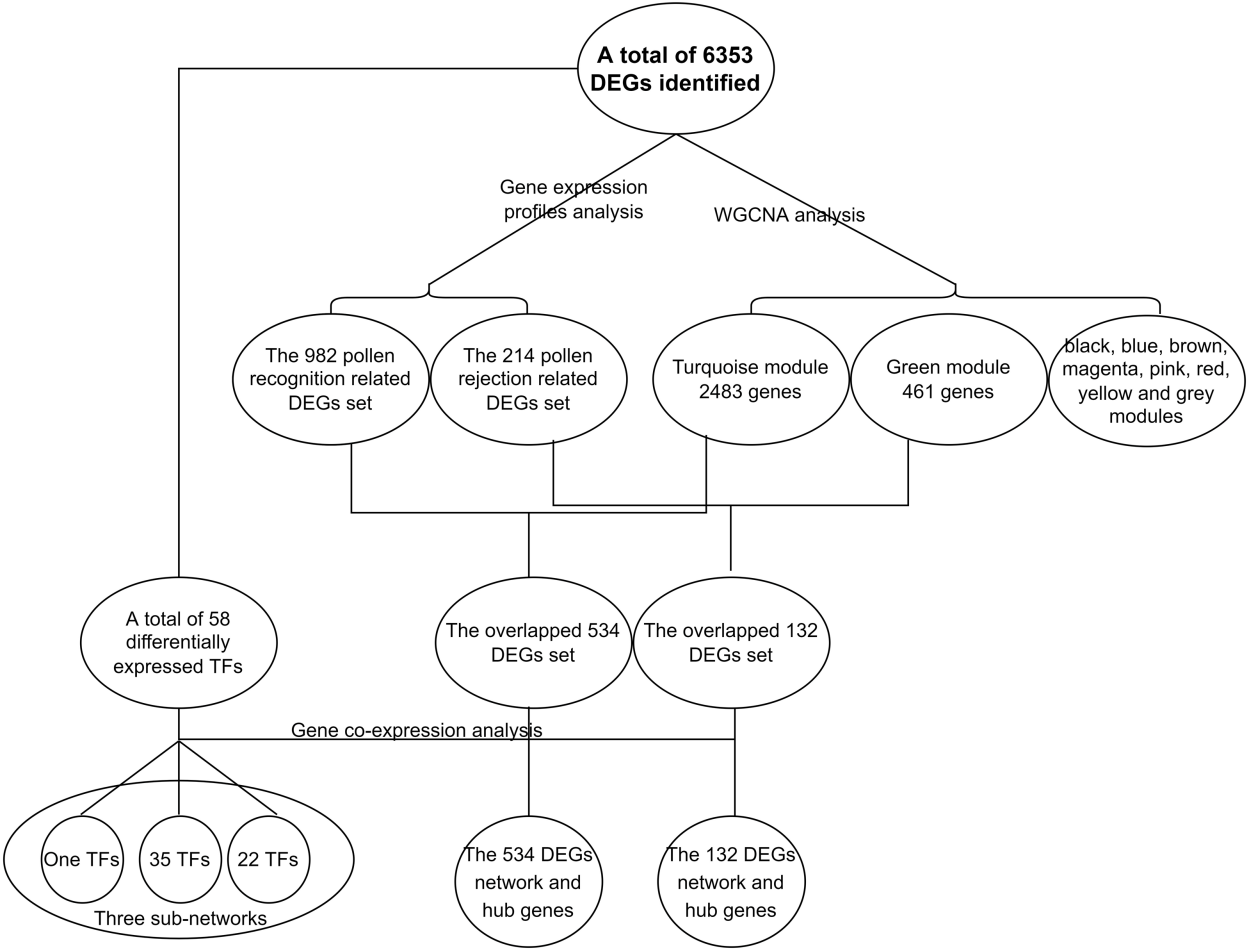
Flowchart of the identificantion of key gene sets.

In the present study, a total of 13 SI-related genes were identified as being non-S locus genes based on the results of gene co-expression analysis, which indirectly supports the validity of this analysis. Based on this, there is a definite possibility that the other hub genes (Tables S7 and S8) from two co-expression networks may also function in some unknown way in the SI mechanism. We also discovered 58 differentially expressed TFs among the DEGs in this investigation. Through the building of a co-expression network for the 58 TFs, we observed that these TFs clustered into three groups, with the first group having only one member (TF1) and the other two groups having 22 (TF22) and 35 (TF35) members, respectively (Fig. S7). Furthermore, the top 50 hub genes of the 534 DEGs network were fully included in the TF22 sub-network, which had a strong correlation with 13 of the 22 TFs (Fig. 5). However, we must point out that 2 of the 13 TFs are also the top 50 hub genes of the 534 DEGs. The workflow diagram of our candidate gene set screening is illustrated in Fig. 6.

The different expressions of the 13 TFs at different time points are depicted in Fig. S8. These TFs were annotated as *YABBY4* (c30021_f1p0), *ANL2* (c105690_f1p0), *OBF1* (c28526_f6p1, c99327_f1p2), *ERF43* (c64391_f1p0), *ARF2* (c105910_f1p1), *BLH* 7 (c129877_f1p4), *KNAT6* (c42568_f1p1), *PIF3* (c166445_f1p1), *HY5* (c36498_f1p1, c71338_f1p1) and *LHY/CCA1* (c125807_f4p5, c137898_f1p7).

They could also potentially be the non-S locus genes of GSI in dragon fruit. This particularly applies to *ERF43* (Su et al., 2020), *ARF2* (Tantikanjana & Nasrallah, 2012), *BLH7,* and *KNAT1* (Arnaud & Pautot, 2014), which have already been found to mediate SI responses in *Brassica rapa, Ipomoea cairica,* and *Theobroma cacao*.

## Discussion

SI has a significant impact on dragon fruit production costs and quality. It plays a significant role in dragon fruit production. It has gotten much attention recently but remains understudied. Therefore, we studied the pollen tube extension in response to self and cross-pollination in a SI dragon fruit variety and variation in gene expression during these two processes by applying a comparative transcriptomics approach.

According to our experimental results and bioinformatic analyses, we believe that dragon fruit displays an S-RNase-based GSI system for three reasons. First, our pollen tube extension experiment results depicted that self-pollinated pollen tubes stopped extension 24 h after pollination and reached only 50% of pistil length, thus confirming that dragon fruit displays a GSI system (McFadden et al., 1992). Second, our data demonstrated that the female determinant of dragon fruit is a glycoprotein. This finding is consistent with the fact that the female S factor of the other known SI systems, S-RNase-based SI and *P. rhoeas* type SI system, are all glycoproteins. Third, we have identified six S-RNase and 158 SLF homologous genes, but no *P. rhoeas* male S homologous genes were identified. The above three arguments strongly suggest the possibility of S-RNase-based GSI in dragon fruit while excluding the *P. rhoeas* type of GSI function in dragon fruit (Sun et al., 2018). Since the S-RNase-based GSI system is the ancestoral SI system in multiple species (Zhao et al., 2021), the finding of S-RNase and F-box would not be sufficient information for finding more detailed and specific mechanisms of the SI type in dragon fruit. The preceding conclusion is based on the fact that all known GSI female S factors are either S-RNase or PrsS type homologs (Zhao et al., 2021). Therefore, based only on these results, one cannot rule out the possibility of an entirely new and unknown SI system in dragon fruit or a more complex than primary S-RNase GSI mechanism, which could involve additional players. Therefore, we set out to explore this possibility. According to recent studies, S-RNase-based SI is the ancestoral SI system commonly found in dicotyledons (Zhao et al., 2021; Lv et al., 2022). During evolution, the S-RNase system may lose its function, making it possible to evolve new SI systems, but the corresponding loci will still be present, such as the *P. rhoeas* type SI, which is controlled by PrsS and PrpS but is still present by non-functional S-RNases.

Our results suggest that dragon fruit displays an S-RNase-based GSI system, which should be controlled by one S-RNase and one or a set of SLF/SFBBs. However, dragon fruits may possess a unique GSI mechanism. One of the unique sides of dragon fruit SI is that its characteristic phenotypic displays the offspring segregation ratio of SI and SC hybridized combination, which is different from the published literature (Zhao et al., 2021). Our data indicated that the segregation ratio from four cross combinations ranges from 4.9% to 7.8% (Table 2), which does not match the expected Mendelian ratios (SI: SC =1:1/0:1) for single locus segregation. This strongly suggests either a much more complex regulatory network or the existence of a distinct SI mechanism in dragon fruit.

**Table 2:** The segregation ratio of self-compatible and incompatible progenies from various cross combinations.

Combinations (♀ × ♂)	Number of SC progeny/ F1 hybrid progeny tested (ratio)
HSJ × DH	5/71 (7.04%)
HSJ × BR	6/96 (6.25%)
QP × DH	4/81 (4.93%)
DH × BR	8/103 (7.84%)

**Notes.**

HSJ, DH, BR and QP in the table are four of our germplasm resources, HSJ and QP are self-incompatibility (SI) varieties, DH and BR are self-compatibility (SC) varieties.

Our co-expression network analysis also uncovered multiple genes which are tightly connected to self-incompatibility, as most of them can be proved to be involved in SI based on homology to previously published genes (Ma et al., 2015; Zhang et al., 2017), suggesting the possibility of the existence of a novel mechanism. We must point out that the SI process is considered a continuous dynamic process (Zhang et al., 2018). In our experimental setup, we started the analysis by dividing the SI process into recognition and rejection. On the one hand, this approach might lead to some information loss. On the other hand, it is superior as it makes the data easier to understand and allows for more accurate results. Based on this idea, we isolated two gene sets from the analysis of gene expression profiles, the 982 DEGs and the 214 DEGs. In our further analysis, we found two other gene sets, the turquoise module and the green module, which overlap with the 982 and the 214 DEGs (Table S5). Overlapping these genes generated two new gene sets, the 534 and the 132 DEGs (Table S5), which were validated by different methodologies to be more accurate and narrow the range of SI-related gene sets. In GO enrichment analysis, we found that the green module and the 214 DEGs gene set shared 44 of the significantly enriched GO terms (the 214 DEGs genes set had 71 and the green module had 75 significantly enriched GO terms), the turquoise module and the 982 DEGs genes set shared 136 of the significantly enriched GO terms (the 982 DEGs genes set has 219 and turquoise module has 373 significantly enriched GO terms).

In the co-expression analysis, 44 of the first 50 hub genes were shared between the co-expression network of the 214 DEGs gene set and the green module (Table S10). The turquoise module and the 982 DEGs gene set shared 27 of their first 50 hub genes (Table S11). This suggests that they not only have a large proportion of genes overlapped. More importantly the overlapped genes are all SI related. Based on this idea, we focused on the two overlapping gene sets and conducted a co-expression analysis. Among them, the 534 DEGs are related to pollen recognition, while the 132 DEGs are related to pollen rejection. However, the annotation of the respective top ten hub genes demonstrated that their demarcation is not that apparent. Both networks have one hub gene annotated as 120 kDa proteins. The seventh hub gene of the 132 DEGs networks is an arabinogalactan protein, an interactor between the pollen tube and female tissues, and a recognition factor rather than a rejection factor. It could be because of the diversity of gene functions or the bias resulting from the bioinformatics analysis.

We believe that our results are significant as most hub genes can be proven to be SI-players based on these genes’ homology to other genes previously reported (Tables S7 and S8). However, the three hub genes from the 132 DEGs network were found not to have a known relationship with SI, and the four hub genes from the 534 DEGs network have no annotation, which is an important issue for future research. Another intriguing observation is that 13 transcription factors (TFs) from the gene set TF22 are highly correlated with the top 50 hub genes from the 534 DEGs network and clustered together unusually. While the other gene sets, the TF35 set, and the ungrouped gene (TF1), were disassociated with the identified hub genes. Unlike the WGCNA or gene expression profiles studies, the co-expression of the TFs is a different analysis employing a novel process, yet the results depict that they are highly correlated.

The TF and hub gene analyses complement and confirm each other, implying a high confidence level in the analytical results. Among the 13 TFs, *YABBY4*, the second hub gene of the network IS534, was previously reported to govern pistil elongation in tomatoes (Massonnet et al., 2020). However, since the pistil length has been fixed in our experiment, it is reasonable to speculate that it has some undiscovered mechanisms in regulating plant reproduction. Furthermore, *YABBY4* was found using two different methodologies, implying that it may play a key role in self-incompatibility.

Exogenous hormones, primarily ethylene and auxin, have been known to help several species overcome SI (Su et al., 2020). In accord with that, four of the 13 TFs were previously implicated in auxin or ethylene regulation (Sood et al., 1982; Dharmasiri et al., 2005; Parry & Estelle, 2006; Tantikanjana & Nasrallah, 2012; Su et al., 2020), suggesting that ethylene and auxin may play a role in dragon fruit SI. However, more data using different scientific approaches will be required to confirm it.

The two gene sets, the overlapped 534 and 132 DEGs, and the 13 differentially expressed TFs are not all self-incompatibility-associated genes. They are solely the sets of candidate genes retrieved after narrowing them down step by step using different methods. Based on the connectivity in the co-expression network, we can rank the importance of each gene according to the connectivity. Thus, we also provide a pool of genes that could be good candidates for study in the future to determine their role in self-incompatibility mechanisms in dragon fruit and other species. Using a combination of several different bioinformatics methods, such as WGCNA, analysis of gene expression profiles, and GO and KEGG enrichment, however, provided the accuracy of the bioinformatics results and conclusions.

This study’s fundamental goal was to explore dragon fruit’s self-incompatibility. Our data provided several new concepts and suggested new paths for future studies, thus providing the foundation for a deeper understanding of self-incompatibility in dragon fruit.

## Conclusions

In this study, we confirmed that dragon fruit exhibited GSI and demonstrated that it operates *via* S-RNase. Using bioinformatic approaches, we also identified relevant candidates’ S genes. In addition, we discovered some non-S locus genes associated with dragon fruit self-incompatibility. Our finding also demonstrated that self-incompatibility in dragon fruit has its intra-species specificity. Overall, our work establishes a solid foundation that will serve as a valuable resource for future detailed studies of the mechanism of self-incompatibility in dragon fruit.

##  Supplemental Information

10.7717/peerj.14165/supp-1 Figure S1 Principal component analysis (PCA) of the repeatability of cross-pollination (CP) and self-pollination (SP) biological replicates at different time points

10.7717/peerj.14165/supp-2 Figure S2 E-value distribution of BLAST hits for the matched unigene sequences, using an E-value cutoff of 1.0E^−5^

10.7717/peerj.14165/supp-3 Figure S3 Result of power analysis graph of the sample size required for given parameters (power = 0.9, two-tailed ANOVA test with *α* = 0.05, sequencing depth = 114)

10.7717/peerj.14165/supp-4 Figure S4 Weighted gene co-expression network analysis (WGCNA) of DEGs A. Analysis of the scale-free fit index for various soft-thresholding powers, the red line indicates the appropriate scale-free topology fit index at 0.9. B. DEGs cluster and modules generated by WGCNA.

10.7717/peerj.14165/supp-5 Figure S5 GO and KEGG enrichment of green module and the 214 DEGs gene set For GO enrichment, the first circle is the GO term of the first 20 enriched terms. Different colors represent different ontologies (blue for biological process and yellow for molecular function); the second circle is the number of genes in GO term of the background and *Q* value of the enrichment. The more genes, the longer the bar, the smaller the *Q* value, and the redder the color; most of the −log10 (*Q* value) is between 10 and 15; the third circle: enriched gene proportion bar chart in purple, the more genes, the longer the bar; the fourth circle: enrich factor value of each GO term (the number of DEGs is divided by the number of genes in the background in GO term, each grid of the background grid line represents 0.1). For KEGG enrichment, the first 20 enriched pathways were illustrated in the figure; the dot’s size and color represent the gene number and *Q* value enriched in each pathway

10.7717/peerj.14165/supp-6 Figure S6 GO and KEGG enrichment of turquoise module and the 982 DEGs gene set For GO enrichment, the first circle: GO term of the first 20 enriched terms. Different colors represent different ontologies (blue for biological process and yellow for molecular function); the second circle is the number of genes in GO term of the background and *Q* value of the enrichment. The more genes, the longer the bar, the smaller the Q value and the redder the color; most of the −log10 (*Q* value) is between 10 and 15; the third circle: enriched gene proportion bar chart in purple, the more genes, the longer the bar; the fourth circle: enrich factor value of each GO term (the number of DEGs is divided by the number of genes in the background in GO term, each grid of the background grid line represents 0.1). For KEGG enrichment, the first 20 enriched pathways were depicted in the figure; the dot’s size and color represent the gene number and *Q* value enriched in each pathway

10.7717/peerj.14165/supp-7 Figure S7 Gene co-expression network of the differentially expressed 58 TFs It consisted of the 58 TFs and their top 50 correlated genes. Due to the weak correlation between some genes, this network was divided into 3 sub-networks, the TF35 sub-network (left of the panel), the TF22 sub-network (right panel) and the TF1 (lower middle of the panel) sub-network. The three sub-networks consisted of the 35, 22 and one of the 58 TFs (bigger light blue nodes) and their top 50 correlated genes (other smaller nodes from brown to yellow). The color of nodes, from brown to blue, represents the degree of nodes.

10.7717/peerj.14165/supp-8 Figure S8 Gene expression heatmap of the 13 TFs The color from blue to red in the figure represents the expression value from low to high.

10.7717/peerj.14165/supp-9 Table S1 Extension of pollen tube at different time points after pollination

10.7717/peerj.14165/supp-10 Table S2 Preview of the sequence quality for the samples in this study

10.7717/peerj.14165/supp-11 Table S3 The randomly selected sequence and corresponding primers

10.7717/peerj.14165/supp-12 Table S4 Modules classified by WGCNA. The 6353 DEGs were classified into ten modules named with colors

10.7717/peerj.14165/supp-13 Table S5 The members of the four key gene sets and their relationship The green module and the 214 DEG sets have 132 genes shared, while the turquoise module and 982 DEG sets have 534 shared genes. The shared genes by two gene sets have been labeled as “shared”

10.7717/peerj.14165/supp-14 Table S6 The homologous F-box, S-Rnase genes and their sequence of dragon fruit

10.7717/peerj.14165/supp-15 Table S7 Hub genes of the overlapped 534 DEGs co-expression network

10.7717/peerj.14165/supp-16 Table S8 Hub genes of the overlapped 132 DEGs co-expression network

10.7717/peerj.14165/supp-17 Table S9 Sequences of the 4 un-annotated genes

10.7717/peerj.14165/supp-18 Table S10 Top 50 hub genes of co-expression network DF214 and green module In total, 44 genes shared between them have been labeled as “shared”

10.7717/peerj.14165/supp-19 Table S11 Top 50 hub genes of co-expression network LAP982 and turquoise module They share 27 genes in common that have been labeled as “common”

## Abbreviations

SP: self-pollination
CP: cross-pollination
SI: self-incompatibility
SC: self-compatibility
GSI: gametophyte self-incompatibility
SSI: sporophytic self-incompatibility
SLF: S Locus F-box
SFBB: S locus F-box brothers
hap: hours after pollinated
TF: Transcription Factor

## Additional Information and Declarations

### DNA Deposition

The following information was supplied regarding the deposition of DNA sequences: Sequences are available at GenBank: PRJNA801848.
https://www.ncbi.nlm.nih.gov/search/all/?term=PRJNA801848.

### Data Availability

The following information was supplied regarding data availability: The raw data are available in the Supplemental Files.
