# Supplementary figures and images for: Global transcriptome dissection of pollen–pistil interactions induced self-incompatibility in dragon fruit (Selenicereus spp.)
Source: PeerJ. 2022 Nov 1;10:e14165. doi: 10.7717/peerj.14165 (PMC9635355; doi:10.7717/peerj.14165)

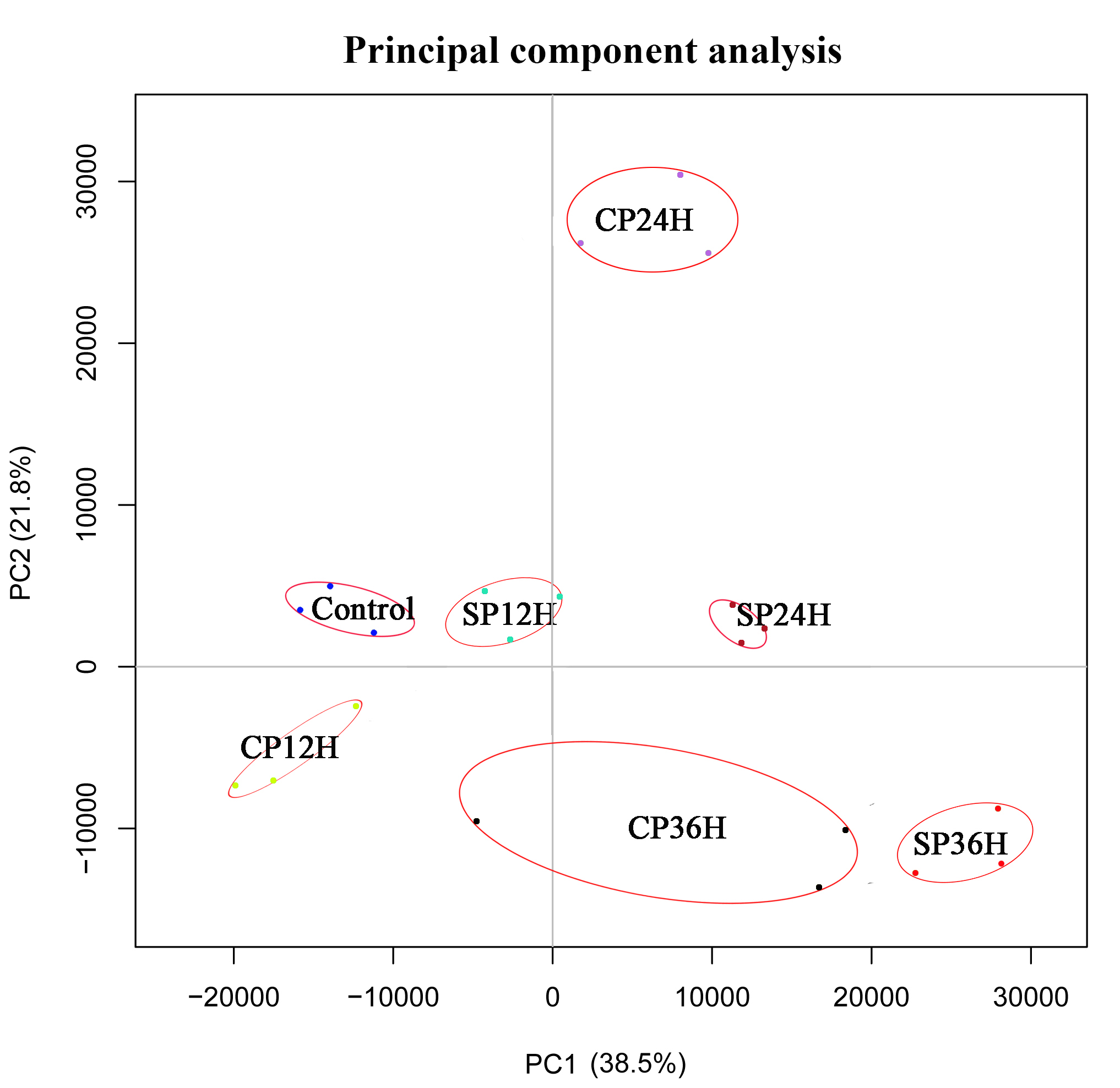

Supplement: Figure S1 [file peerj-10-14165-s001.jpg]

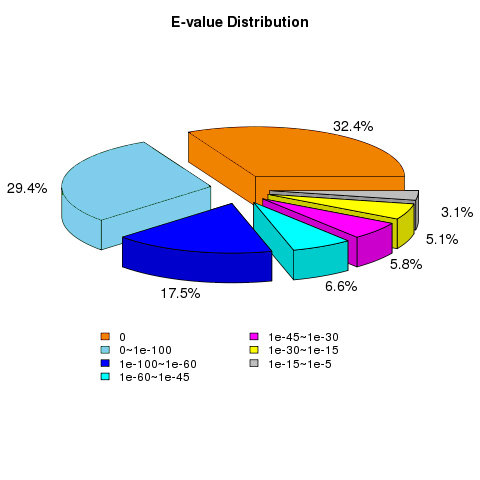

Supplement: Figure S2 [file peerj-10-14165-s002.jpg]

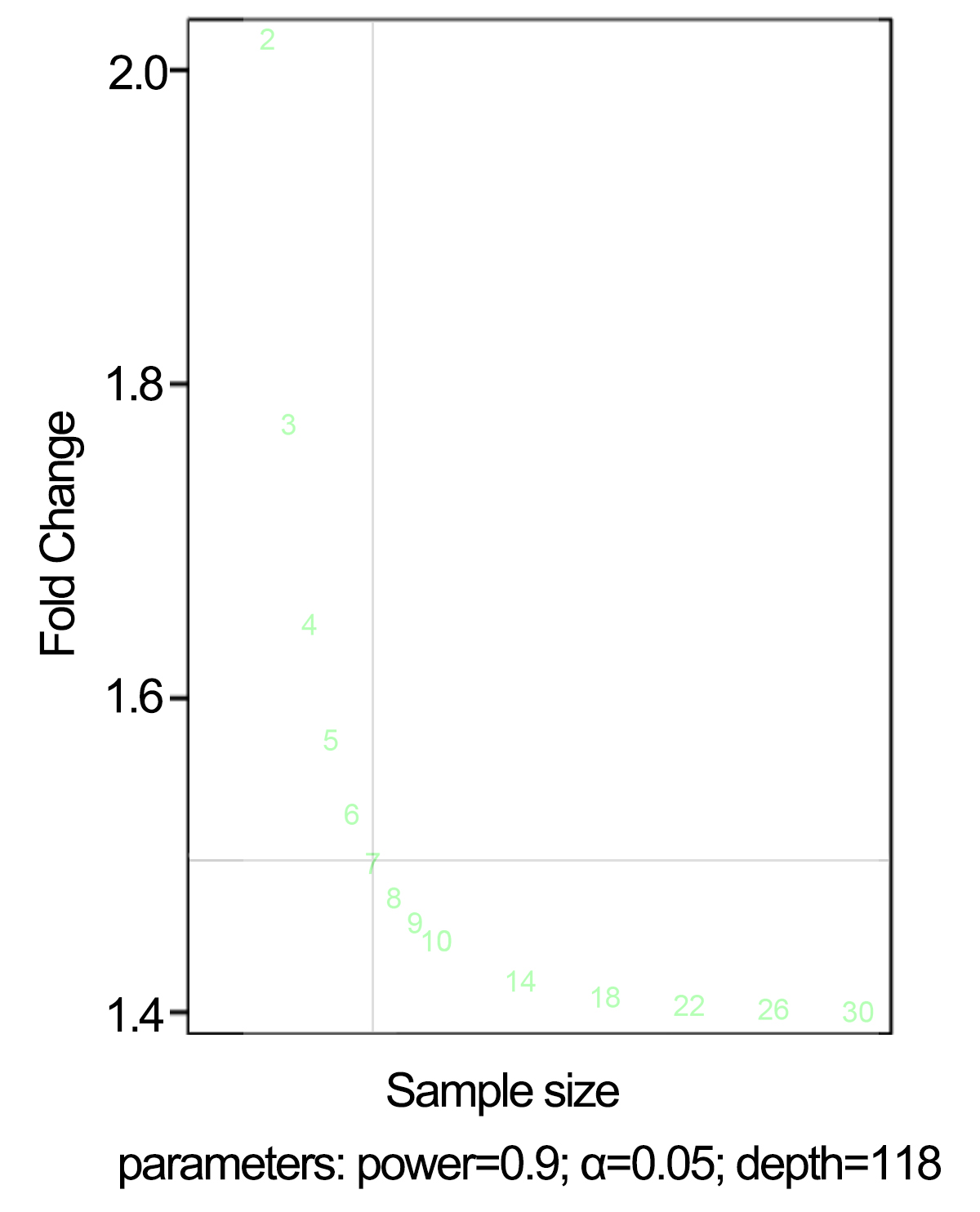

Supplement: Figure S3 [file peerj-10-14165-s003.jpg]

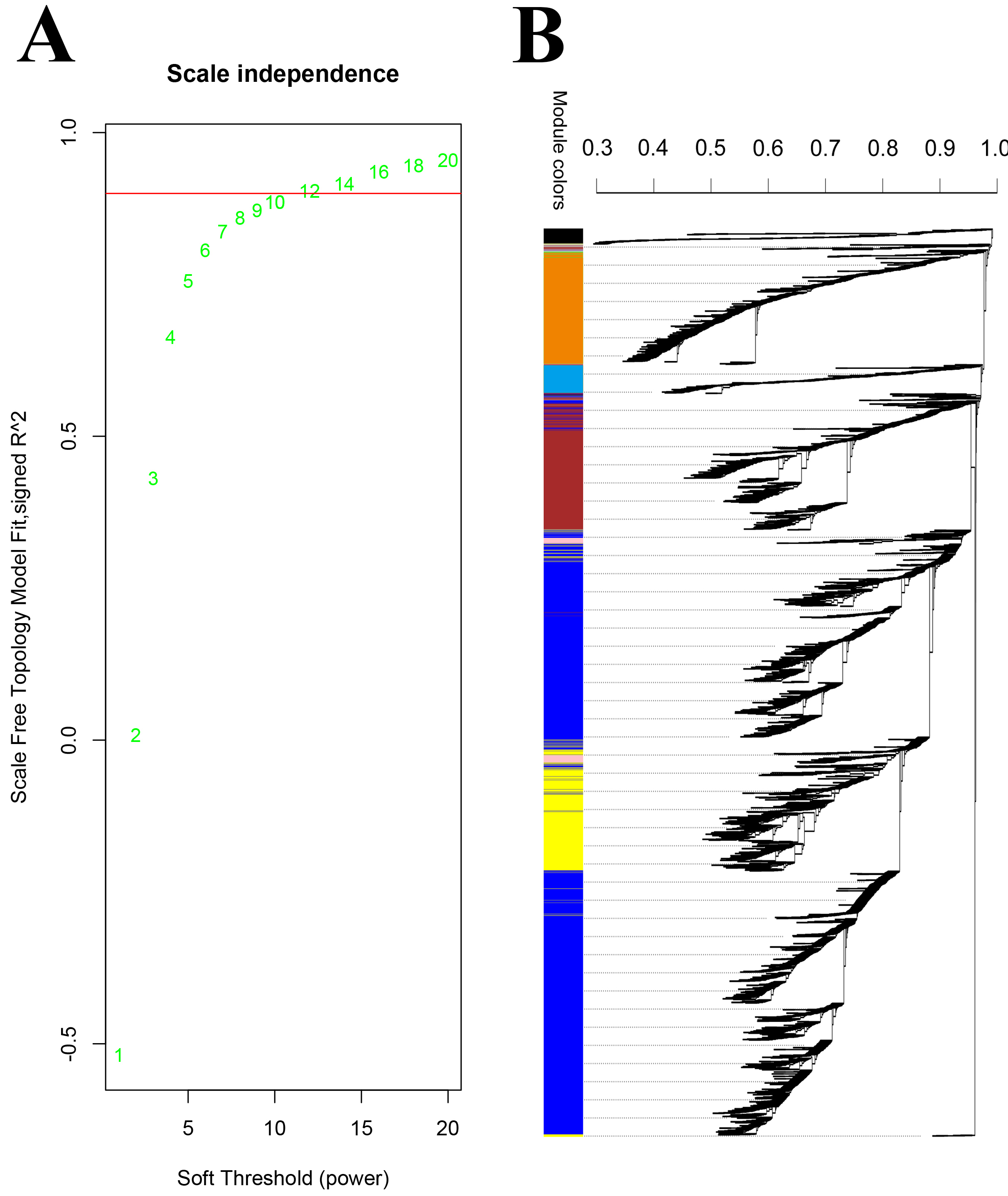

Supplement: Figure S4 — A. Analysis of the scale-free fit index for various soft-thresholding powers, the red line indicates the appropriate scale-free topology fit index at 0.9. B. DEGs cluster and modules generated by WGCNA. [file peerj-10-14165-s004.jpg]

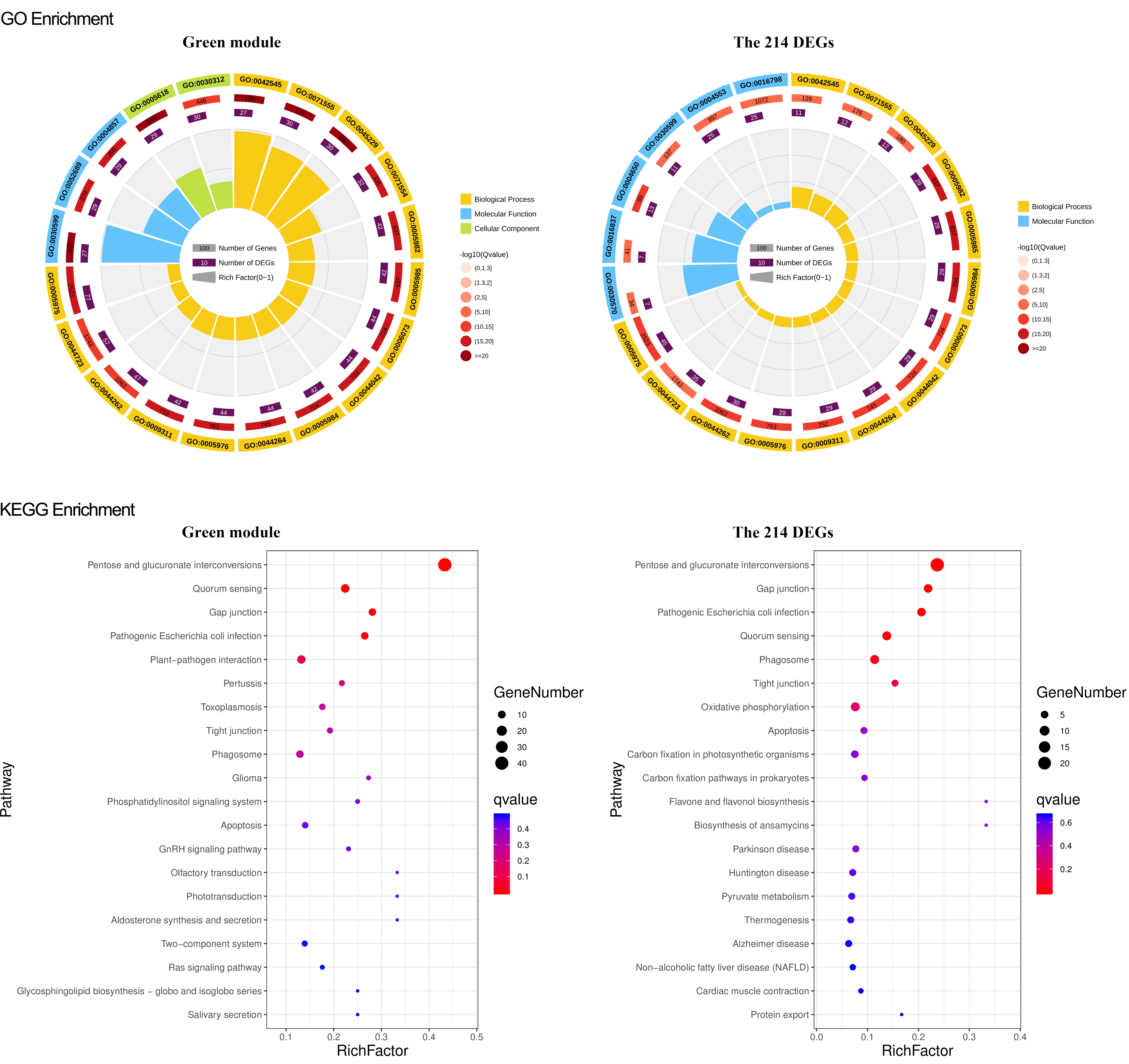

Supplement: Figure S5 — For GO enrichment, the first circle is the GO term of the first 20 enriched terms. Different colors represent different ontologies (blue for biological process and yellow for molecular function); the second circle is the number of genes in GO term of the background and Q value of the enrichment. The more genes, the longer the bar, the smaller the Q value, and the redder the color; most of the −log10 (Q value) is between 10 and 15; the third circle: enriched gene proportion bar chart in purple, the more genes, the longer the bar; the fourth circle: enrich factor value of each GO term (the number of DEGs is divided by the number of genes in the background in GO term, each grid of the background grid line represents 0.1). For KEGG enrichment, the first 20 enriched pathways were illustrated in the figure; the dot’s size and color represent the gene number and Q value enriched in each pathway [file peerj-10-14165-s005.jpg]

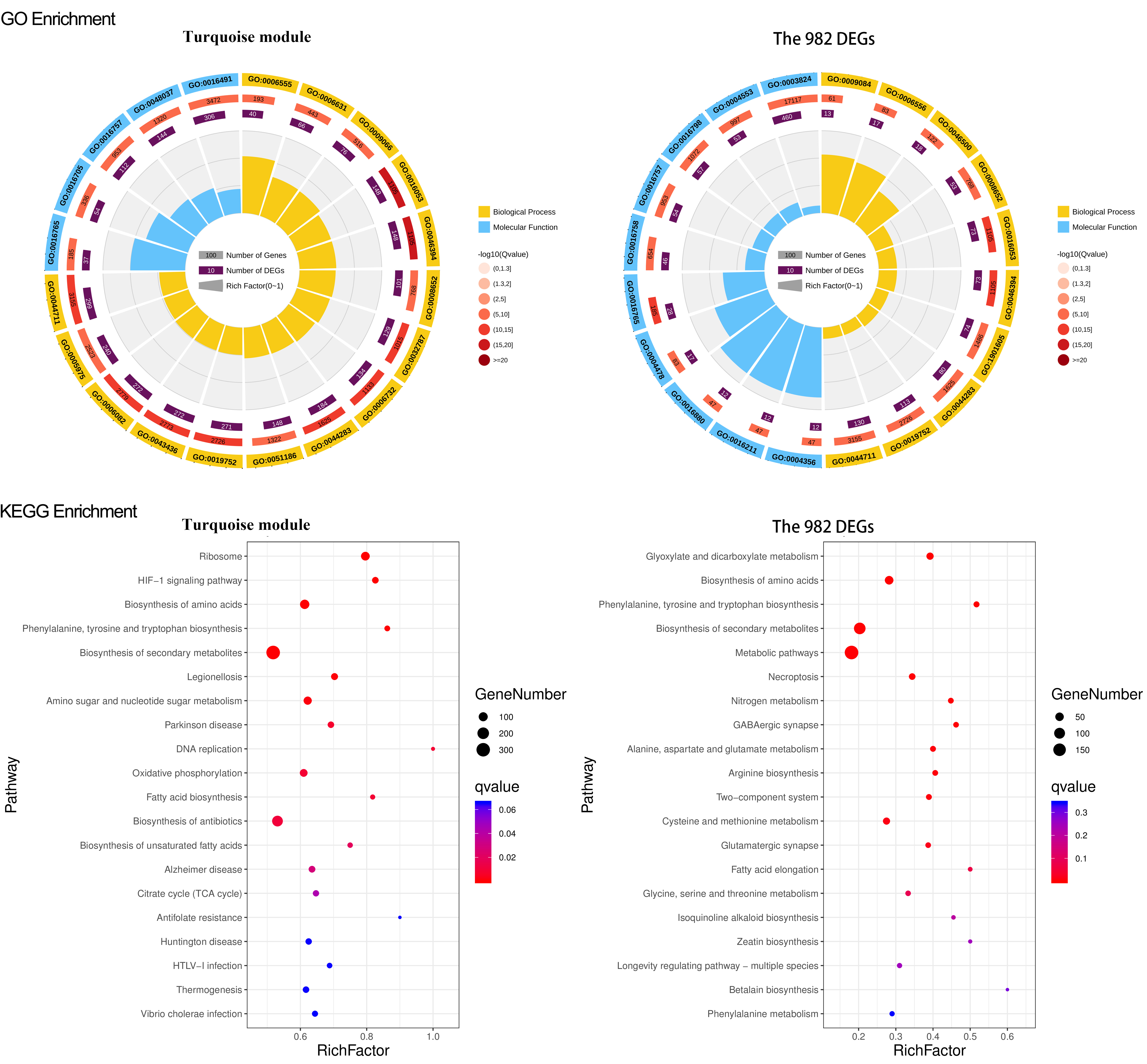

Supplement: Figure S6 — For GO enrichment, the first circle: GO term of the first 20 enriched terms. Different colors represent different ontologies (blue for biological process and yellow for molecular function); the second circle is the number of genes in GO term of the background and Q value of the enrichment. The more genes, the longer the bar, the smaller the Q value and the redder the color; most of the −log10 (Q value) is between 10 and 15; the third circle: enriched gene proportion bar chart in purple, the more genes, the longer the bar; the fourth circle: enrich factor value of each GO term (the number of DEGs is divided by the number of genes in the background in GO term, each grid of the background grid line represents 0.1). For KEGG enrichment, the first 20 enriched pathways were depicted in the figure; the dot’s size and color represent the gene number and Q value enriched in each pathway [file peerj-10-14165-s006.jpg]

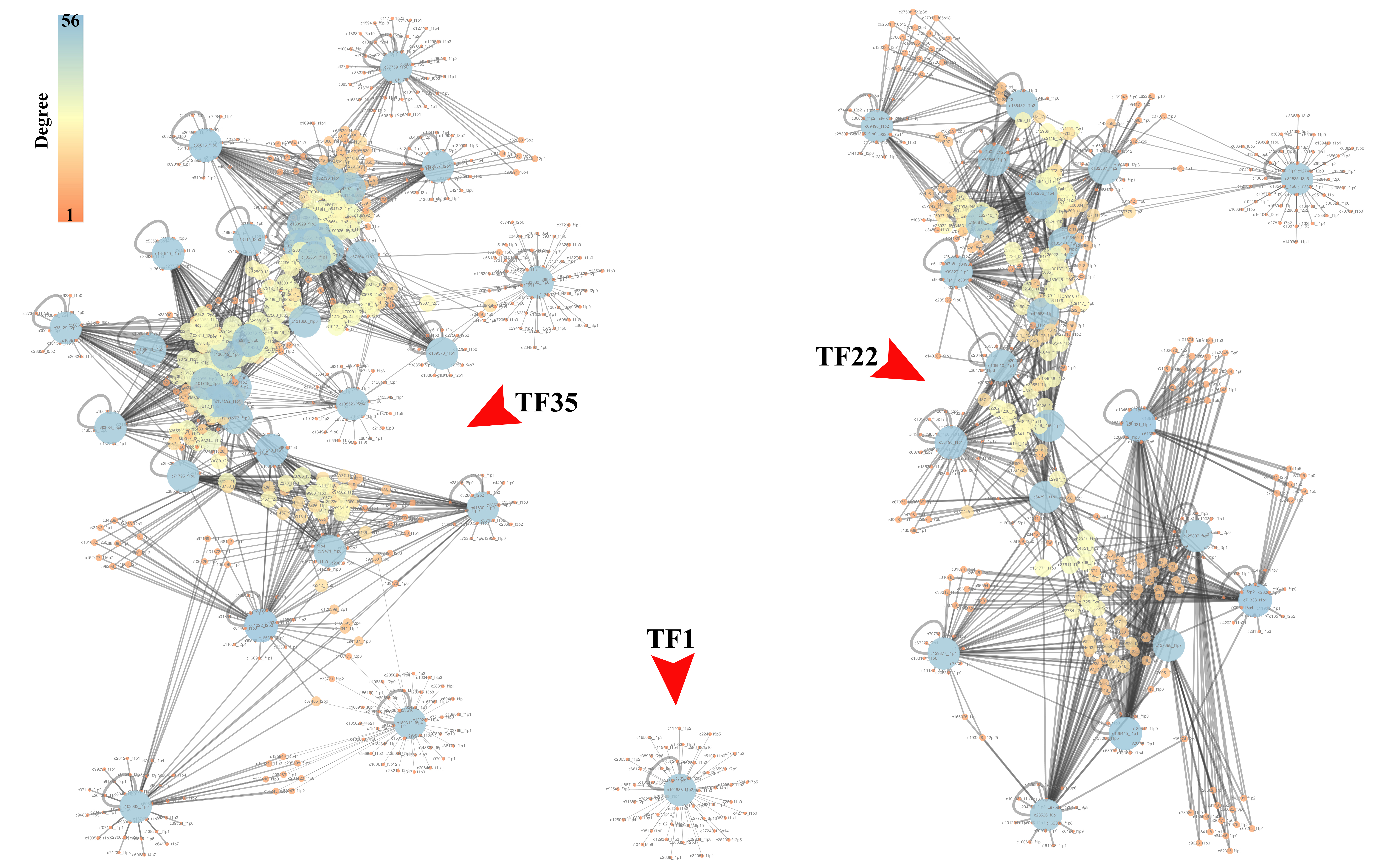

Supplement: Figure S7 — It consisted of the 58 TFs and their top 50 correlated genes. Due to the weak correlation between some genes, this network was divided into 3 sub-networks, the TF35 sub-network (left of the panel), the TF22 sub-network (right panel) and the TF1 (lower middle of the panel) sub-network. The three sub-networks consisted of the 35, 22 and one of the 58 TFs (bigger light blue nodes) and their top 50 correlated genes (other smaller nodes from brown to yellow). The color of nodes, from brown to blue, represents the degree of nodes. [file peerj-10-14165-s007.jpg]

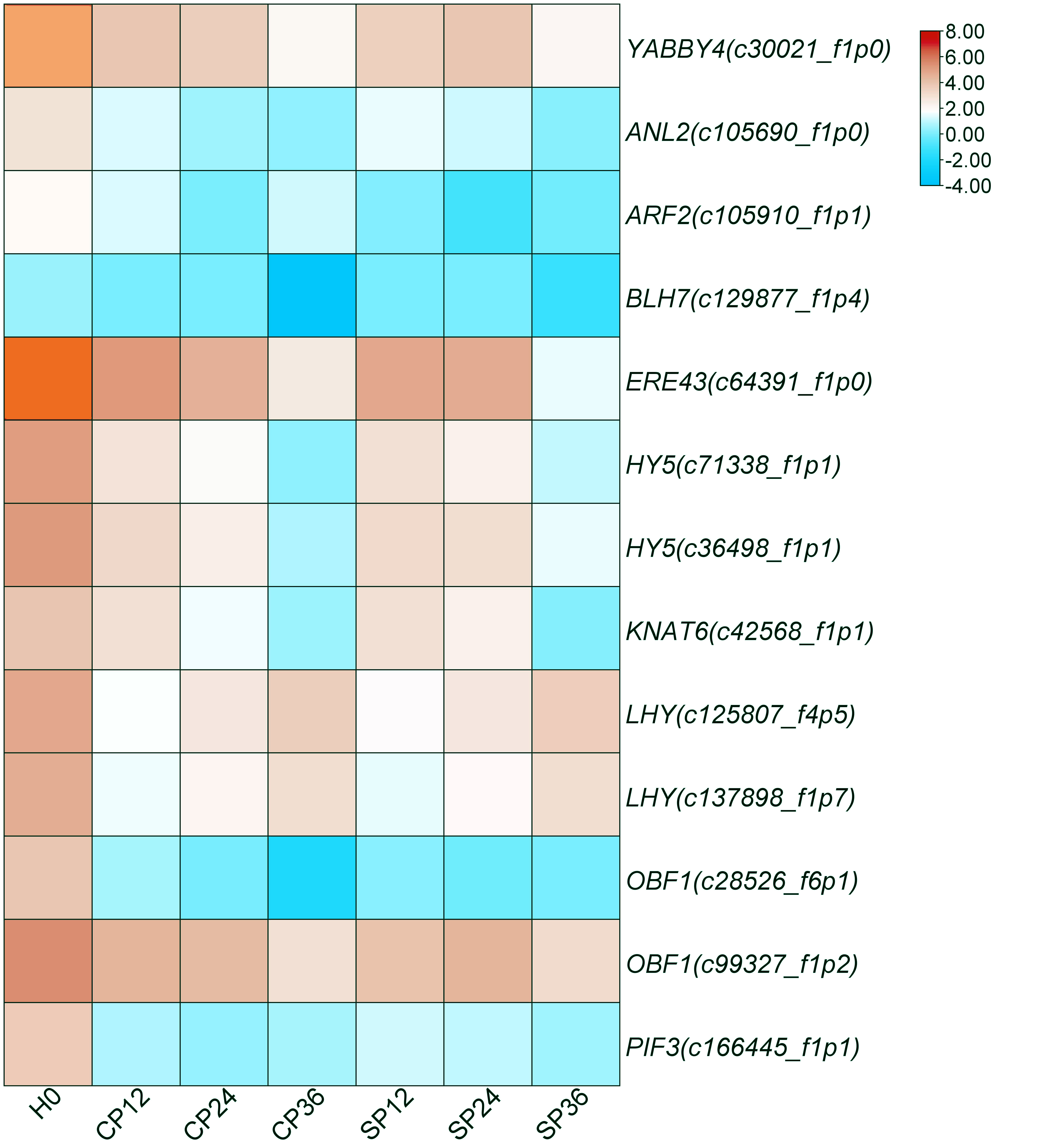

Supplement: Figure S8 — The color from blue to red in the figure represents the expression value from low to high. [file peerj-10-14165-s008.jpg]
